# Severe COVID-19 in pregnancy: evaluation of ventilatory outcomes on a 101-cases cohort

**DOI:** 10.1590/S1678-9946202567051

**Published:** 2025-08-18

**Authors:** Isabel Cristina Melo Mendes, Ana Luiza Martins de Oliveira, Priscila Martins Pinheiro Trindade, Glaucia de Melo Rodrigues, Clarisse Pimentel, Claudia Caminha Escosteguy, Rafael Mello Galliez

**Affiliations:** 1 Universidade Federal do Rio de Janeiro, Programa de Pós-Graduação em Doenças Infecciosas e Parasitárias, Rio de Janeiro, Rio de Janeiro, Brazil; 2 Instituto Estadual de Infectologia São Sebastião, Rio de Janeiro, Rio de Janeiro, Brazil; 3 Universidade Federal Fluminense, Faculdade de Farmácia, Niterói, Rio de Janeiro, Brazil; 4 Hospital Federal dos Servidores do Estado, Rio de Janeiro, Rio de Janeiro, Brazil; 5 Universidade Federal do Rio de Janeiro, Faculdade de Medicina, Rio de Janeiro, Rio de Janeiro, Brazil,; 6 Universidade Federal do Rio de Janeiro, Núcleo de Enfrentamento e Estudos de Doenças Infecciosas Emergentes e Reemergentes, Rio de Janeiro, Rio de Janeiro, Brazil

**Keywords:** COVID-19, Pregnancy, Puerperium, ICU, Ventilation

## Abstract

Pregnant and postpartum women are considered at increased risk for severe COVID-19. However, information about disease progression and management in this population is scarce. This study aims to describe sociodemographic, clinical, and radiological characteristics of pregnant and postpartum women admitted to intensive care due to severe COVID-19, emphasizing respiratory outcomes. This is a retrospective, descriptive cohort study evaluating consecutive admissions of pregnant and postpartum women to an infectious diseases intensive care unit due to confirmed or suspected COVID-19, from May 2020 to June 2022. Numerical variables were described by median and interquartile range (IQR), and categorical variables, by frequency and percentage. Missing data were excluded from the analysis. A total of 101 admissions were recorded (85 pregnant and 16 postpartum women), with most patients in their second or third trimester. Forty-seven women (46.5%) required invasive mechanical ventilation (IMV), most of whom (62.1%) showed at least 50% of lung involvement on CT scans and requiring neuromuscular blocking agents (89.1%). Lethality was 15.8% in the cohort and 34.0% among women who required IMV. Pregnant and postpartum women are at risk of developing severe COVID-19, with high mortality and need for IMV and neuromuscular blocking. They should be prioritized in public health policies addressing COVID-19.

## INTRODUCTION

Since its emergence in 2019, the SARS-CoV-2 pandemic has caused millions of infections and deaths worldwide. Several groups have been identified as being at a higher risk of developing severe disease and experiencing adverse outcomes^1^.

Although earlier studies have not found an association between pregnancy and severe COVID-19^2^, more recent data have demonstrated that pregnant and postpartum women are at increased risk for hospitalization, intensive care, invasive mechanical ventilation (IMV), obstetric complications, and death^3-6^.

However, information on the course and management of the disease—particularly regarding respiratory compromise and support—remains limited, as pregnant and postpartum women are often excluded from most large-scale COVID-19 studies^7^.

This study aims to describe sociodemographic, clinical, and radiological characteristics of pregnant and postpartum women admitted to an intensive care unit (ICU) for confirmed or suspected severe COVID-19, with emphasis on respiratory aspects.

### Ethics

This study was approved by the Research Ethics Committee according to all Ethical in Human Research regulations (process Nº 30161620.0.0000.5257 and 30127020.0.0000.0068). Informed consent was not required due to the retrospective design of the study. The study adhered to the Strengthening of the Reporting of Observational Studies in Epidemiology (STROBE) guidelines.

The data used in this study have sensible information and therefore cannot be made public. Raw unidentified data and codes used will be made available upon request to the corresponding author and after the authors’ approval. All measures to ensure the right of privacy and data security will be taken.

## MATERIALS AND METHODS

### Study design, location, and population

This is a retrospective cohort study evaluating consecutive admissions of pregnant and postpartum women in an ICU specialized in infectious diseases, due to confirmed or suspected COVID-19, from May 2020 to June 2022. Patients were referred from various healthcare units within the same state. Cases were laboratory-confirmed if they had a positive diagnostic test, either RT-PCR or rapid antigen test, on a nasopharyngeal swab sample.

For cases with a negative result, medical records and notification sheets were reviewed to identify previous positive results obtained at the first healthcare unit and to assess the clinical likelihood of COVID-19. Cases with clinical, epidemiological, and radiological characteristics compatible with COVID-19 were included. When there was no history of a positive diagnostic test and an alternative etiology was deemed more likely, cases were considered negative and excluded from the analysis. Follow-up extended from ICU admission to ICU discharge or death.

### Data collection and variables of interest

Data collected included clinical, laboratory, radiological, and sociodemographic information obtained from each patient’s medical and multidisciplinary records.

The following variables were considered of interest:

- Sociodemographic: age and ethnicity;
- Clinical: presence of comorbidities, symptom duration prior to ICU admission, immunization status at admission, laboratory evidence of SARS-CoV-2 infection, hemodialysis, pregnancy status at admission (pregnant or postpartum), gestational age at admission, obstetric complications, length of hospitalization, and outcome (discharge or death);
- Ventilation: need for oxygen support, use of non-invasive ventilation (NIV) and IMV, duration of IMV, time from ICU admission to IMV initiation, prone positioning, tracheostomy, administration of neuromuscular blocking agents (NMBA), duration of NMBA administration, first ventilation mode used, initial positive end-expiratory pressure (PEEP) value, and initial pressure of oxygen/fraction of oxygen (P/F) ratio;
- Radiological: percentage of lung involvement and presence of images other than ground-glass opacities on chest computerized tomography (CT) scans.

Ethnicity was self-reported or defined by the attending physician, following the definition used by the Brazilian Institute of Geography and Statistics (Instituto Brasileiro de Geografia e Estatistica [IBGE]): Black, Mixed-ethnicity, White, Asian, and Indigenous. Age was registered as complete years based on documentation provided at hospital admission. Patients were considered immunized if, at admission, they had received all recommended doses according to the type of vaccine. Partially or unvaccinated women were considered non-immunized. Vaccination status was confirmed according to the records of the National Immunization Program (Programa Nacional de Imunizacao), a national electronic system that tracks all COVID-19 vaccinations in the country.

The seven-category ordinal scale consisted of the following categories: 1) not hospitalized, with resumption of normal activities; 2) not hospitalized, but unable to resume normal activities; 3) hospitalized, not requiring supplemental oxygen; 4) hospitalized, requiring supplemental oxygen; 5) hospitalized, requiring high-flow nasal oxygen therapy, NIV, or both; 6) hospitalized, requiring extracorporeal membrane oxygenation (ECMO), IMV, or both; and 7) death^8^.

Genomic surveillance data from the state were used to determine the predominant SARS-CoV-2 variants. A lineage was considered predominant when its detection rate exceeded 90% of individuals within a given period. Based on these data, predominant variants during the study period were: 1) original lineage, from March to December 2020; 2) P2, from December 2020 to February 2021; 3) Gamma, from March to June 2021; 4) Delta, from August to November 2021; and 5) Omicron, from January to March 2022^9^.

### Statistical analysis

For descriptive analysis, numerical variables were described in median and interquartile range (IQR), while categorical variables were described in frequency and percentage. Missing data were excluded. P-values were estimated with Wilcoxon rank-sum test, Pearson’s chi-squared test, and Fisher’s exact test. Datasets were built using Google Sheet^®^ and Excel^®^, and anonymized data were exported to the R software in .cvs format. All statistical analyses were performed using the R software version 4.2.2 (R Foundation for Statistical Computing).

## RESULTS

A total of 104 admissions occurred during the study period. Of these, three were excluded as they were not considered COVID-19 cases. Therefore, 101 ICU admissions from May 2020 to June 2022 were included and analyzed (85 pregnant and 16 postpartum women). Most women were in the second or third trimester of pregnancy at the time of admission (median gestational age of 29 weeks; IQR = 24–34). The median age was 30 years (IQR = 25–34). The median time from symptom onset to ICU admission was 9 days (IQR = 6–11), and the median ICU length of stay was 8 days (IQR = 4–21).

Among the 85 women admitted while pregnant, 33 pregnancy terminations occurred during hospitalization. A total of 61 obstetric complications were recorded, with preterm birth being the most frequent, followed by hypertensive disorders of pregnancy. There were eight stillbirths/fetal losses among women who were pregnant at the time of admission and two neonatal deaths among infants born to women who delivered while in the ICU.

Regarding oxygen support, eight (7.9%) women were admitted already on IMV and 55 (54.5%) were receiving another form oxygen support (26 on low-flow nasal cannula and 29 on non-rebreather mask). Forty-seven (46.5%) women required IMV at some point during hospitalization, either at ICU admission or later. Among those not already intubated upon admission, the median time from ICU admission to orotracheal intubation was 2.5 days. Table 1 shows the characteristics of the included patients.

**Table 1:** Characteristics of patients admitted to the ICU.

Characteristic	Overall (N = 101)	No IMV (N = 54)	IMV (N = 47)	p-value
Age	30 (IQR = 25–34)	29 (IQR = 25–33)	31 (IQR = 26–35)	0.3
Ethnicity				0.011
White	41/83 (49%)	13/38 (34%)	28/45 (62%)	
Non-white	42/83 (51%)	25/38 (66%)	17/45 (38%)	
Missing	18	16	2	
Pregnancy status				0.013
Postpartum	16/101 (16%)	4/54 (7.4%)	12/47 (26%)	
Pregnant	85/101 (84%)	50/54 (93%)	35/47 (74%)	
Gestational age at admission	29 (IQR = 24–34)	27 (IQR = 21–33)	29 (IQR = 25–34)	0.2
Missing	18	6	12	
Gestational trimester at admission				0.3
First trimester	3/83 (3.6%)	3/48 (6.2%)	0/35 (0%)	
Second trimester	38/83 (46%)	23/48 (48%)	15/35 (43%)	
Third trimester	42/83 (51%)	22/48 (46%)	20/35 (57%)	
Duration of symptoms until admission (days)	9 (IQR = 6–11)	9 (IQR = 5.5–12.5)	8 (IQR = 6–10)	0.6
Missing	7	3	4	
Number of comorbidities				0.5
0	49 (50.0%)	29 (55.0%)	20 (44.0%)	
1	25 (26.0%)	13 (25.0%)	12 (27.0%)	
2	13 (13.0%)	5 (9.4%)	8 (18.0%)	
3	9 (9.2%)	4 (7.5%)	5 (11.0%)	
4	2 (2.0%)	2 (3.8%)	0 (0.0%)	
Missing	3	1	2	
7-category ordinary scale (WHO)				<0.001
3	26/88 (29.5%)	21/46 (45.7%)	5/42 (11.9%)	
4	26/88 (29.5%)	20/46 (43.5%)	6/42 (14.3%)	
5	29/88 (33.0%)	5/46 (10.9%)	24/42 (57.1%)	
6	7/88 (8.0%)	0/46 (0.0%)	7/42 (16.7%)	
Missing	13	8	5	
Immunization status				0.027
Non-immunized	82/88 (93.1%)	40/46 (87.0%)	42/42 (100.0%)	
Immunized	6/88 (6.8%)	6/46 (13.0%)	0/42 (0.0%)	
Missing	13	8	5	
Thorax CT scan performed	45/101 (45.0%)	22/54 (40.7%)	23/47 (48.9%)	0.4
Proportion of pulmonary compromise (%)				0.0065
< 25	3/37 (8.1%)	3/17 (17.6%)	0/20 (0.0%)	
25-50	11/37 (29.7%)	7/17 (41.2%)	4/20 (20.0%)	
50	8/37 (21.6%)	4/17 (23.5%)	4/20 (20.0%)	
50–75	10/37 (27.0%)	2/17 (11.8%)	8/20 (40.0%)	
>75	5/37 (13.5%)	1/17 (5.9%)	4/20 (20.0%)	
Missing	64	37	27	
Hemodialysis	12/100 (12.0%)	0/53 (0.0%)	12/47 (25.5%)	<0.001
Missing	1	1	0	
Length of ICU hospitalization (days)	8 (IQR = 4–21)	5 (IQR = 3–7)	21 (IQR = 12–38)	<0.001
Obstetric complications	41/56 (73.2%)	9/24 (37.5%)	32/32 (100.0%)	<0.001
Missing	45	30	15	
Death	16/101 (16%	0/54 (0%)	16/47 (34%)	<0.001

IMV = invasive mechanical ventilation; IQR = interquartile range; WHO = World Health Organization; CT = chest tomography; ICU = intensive care unit.

Regarding radiological findings, 37 women had pulmonary CT scans results registered. Most (62.1%) showed at least 50% of compromised lung parenchyma with ground-glass opacities, which was considered suggestive of viral pneumonia. Consolidations were also prevalent (Table 2).

**Table 2:** Description of other images, besides ground-glass opacities, found in chest tomography scans.

Presence of other images	n/N (%)
No	17/37 (45.9%)
Consolidation	16/37 (43.2%)
Pleural effusion	4/37 (10.8%)
Bronchiectasis	1/37 (2.7%)
Atelectasis	1/37 (2.7%)

Among the group of women who required IMV, the most commonly used initial ventilation mode—i.e., the first ventilation mode considered adequate by the attending physicians—was pressure-controlled (70.0%), followed by volume-controlled (25.0%). The mean initial PEEP was 12 cmH_2_O. The median PO2/FiO2 on the first day of IMV was 209. Most women (89.1%) required NMBA, which were administered for a median duration of 6 days (IQR = 3-13).

Twenty women (44.4%) underwent prone positioning at least once, and 13 (27.7%) were tracheostomized due to prolonged time in invasive ventilation. The median duration of IMV was 13 days (IQR = 6-24). Twenty women of the 47 who required IMV had pulmonary CT scan results registered. Of these, 40.0% had an estimated lung involvement of 50% to 75%.

There were 16 deaths in the cohort, corresponding to a mortality of 15.8%. All women who died were in IMV. Among the subgroup of women who required IMV, lethality was 34.0%. Table 3 describes the characteristics of women who required IMV. Compared with the group of pregnant women, mortality was higher among women admitted during postpartum (12.9% [11/85] versus 31.2% [5/16], respectively).

**Table 3:** Characteristics of patients that required invasive mechanical ventilation.

Characteristic	N = 47 (p25, p75, or %)
7-point ordinal severity scale (WHO)	
3	5/42 (11.9%)
4	6/42 (14.3%)
5	24/42 (57.1%)
6	7/42 (16.7%)
Missing	5 (10.6%)
Proportion of pulmonary parenchyma compromise (%)	
25–50	4/20 (20.0%)
50	4/20 (20.0%)
50–75	8/20 (40.0%)
>75	4/20 (20.0%)
Missing	27 (57.4%)
Initial ventilatory mode	
Pressure-controlled ventilation (PCV)	28/40 (70.0%)
Pressure-regulated volume control (PRVC)	2/40 (5.0%)
Volume-controlled ventilation (VCV)	10/40 (25.0%)
Missing	7 (14.9%)
Initial PEEP	
8	6/40 (15.0%)
9	1/40 (2.5%)
10	12/40 (30.0%)
12	5/40 (12.5%)
14	5/40 (12.5%)
16	8/40 (20.0%)
18	2/40 (5.0%)
20	1/40 (2.5%)
Missing	7 (14.9%)
Days in IMV	13 (6, 24)
P/F ratio on D1 of IMV	209 (158, 267)
Use of neuroblocking drugs	41/46 (89.1%)
Missing	1 (2.1%)
Days of neuroblocking use	6 (3, 13)
Prone position maneuver	20/45 (44.4%)
Missing	2 (4.2%)
Tracheotomy	13/47 (27.7%)
Death	16/47 (34.0%)

WHO = World Health Organization; PEEP = positive end-expiratory pressure; IMV = invasive mechanical ventilation; P/F = pressure of oxygen/fraction of oxygen.

## DISCUSSION

In this cohort, severe COVID-19 in pregnant and postpartum women was associated with high mortality (15.8%), especially among those who required advanced oxygen support. Most women in IMV required NMBA and elevated PEEP levels (≥12 cmH2O) to maintain satisfactory ventilation.

Our results are consistent with findings from other studies. A descriptive study conducted in the United States, which analyzed 64 pregnant or postpartum women with severe or critical COVID-19, found a difference of about 7 days from symptom onset to hospital admission, and a median length of stay of 6 days. The same study also found a high intubation rate among women with critical COVID-19 (95.0%), defined as respiratory failure requiring mechanical ventilation, septic shock, or multiple organ dysfunction or failure^10^. These findings are similar to those found in our study, showing a predominance of hospitalizations during the second week of illness.

Significant lethality among postpartum women has also been found in other studies. A prospective cohort study conducted in Brazil, evaluating COVID-19 cases during pregnancy, found a six-fold increase in the risk of severe acute respiratory syndrome (SARS) in postpartum women compared to those in the first trimester^11^. Similarly, COVID-19 was associated with a higher risk of preterm birth and severe neonatal morbidity^11^.

Use of NMBA and prone positioning was elevated in our cohort, with higher frequencies than those reported in studies conducted in other countries. Cohorts evaluating pregnant and postpartum women admitted to ICU with severe COVID-19 reported prone positioning rates of 6.0% to 34.4%, and neuromuscular blockade use of 28.1%^10,12^—much lower than those of our cohort (44.4% and 89.1%, respectively). Notably, the cited studies reported no deaths, contrasting with our mortality rate of 15.8%.

Differences in resource availability may contribute to the differences found, as certain COVID-19 treatments and technologies—such as extracorporeal membrane oxygenation (ECMO)—vary widely across Brazil, especially within public healthcare units^13^. This may also include unequal access to adequate levels of care, such as ICU beds and specialized support. Data from the Obstetric Observatory Brazil (OOBr) COVID-19 project showed that, from 2020 to 2021, 22.5% of pregnant or postpartum women who died from COVID-19 did not have access to an ICU, and 33.5% did not receive invasive ventilatory support^14^. Moreover, data on the efficacy and pharmacokinetics of recommended drugs—such as remdesivir, tocilizumab, and baricitinib—in pregnant women remain scarce^15,16^. No women in our study received antivirals or anti-inflammatory drugs, apart from corticosteroids, as treatment for COVID-19.

Another Brazilian study, involving pregnant and postpartum women admitted to an ICU due to COVID-19, also registered a 0.0% maternal mortality^7^. The median time from symptom onset to ICU admission (10.0 days), IMV incidence (55.2%), median IMV duration (16.5 days), median PEEP (11 cmH2O), and median ICU length of stay (14.0 days) were similar to ours, although IMV incidence and median hospitalization time were lower in our study (9.0 days, 46.5%, 13.0 days, 12 cmH2O, and 8.0 days, respectively). However, NMBA administration (51.7%) was less frequent than in our cohort (89.1%).

These differences may be due to a more severe disease course among our patients. It is important to note that the United States studies were conducted in 2020, and the Brazilian study, from June 2020 to August 2021; our study covered admissions from 2020 to 2022. Therefore, our cohort included a wider range of SARS-CoV-2 variants with differing virulence. According to genomic surveillance data, during our study period, the original lineage, as well as the P2, Gamma, Delta, and Omicron variants, circulated in Rio de Janeiro^9^. The Delta variant is associated with higher virulence and mortality rates^17-19^, and our study is the only one to include data of when it was the predominant variant. However, the small number of cases during each wave precluded comparative analysis among variants.

Our study has several limitations. First, as a descriptive study based on medical records, it is subject to misclassification and varying degrees of missing data. We searched multiple records to verify classifications and minimize missing data. Nonetheless, we cannot rule out misclassification. Second, the relatively small sample size may limit generalization. Although our cohort includes patients referred from multiple cities across the state, which likely reduces selection bias and enhances representativeness, the findings may not represent all pregnant and postpartum women.

## CONCLUSIONS

Pregnant and postpartum women with COVID-19 are at increased risk of developing severe disease, with extensive lung involvement and often requiring IMV, neuromuscular blocking, and prone positioning. This population should be prioritized in public health policies addressing COVID-19, emphasizing vaccination, early referral, and improved availability of medical resources, such as ICU beds.
